# The association between dietary selenium intake and diabetes: a cross-sectional study among middle-aged and older adults

**DOI:** 10.1186/s12937-015-0007-2

**Published:** 2015-02-18

**Authors:** Jie Wei, Chao Zeng, Qian-yi Gong, Hao-bin Yang, Xiao-xiao Li, Guang-hua Lei, Tu-bao Yang

**Affiliations:** 1 Department of Epidemiology and Health Statistics, School of Public Health, Central South University, Changsha, Hunan Province 410008 China; 2 Department of Orthopaedics, Xiangya Hospital, Central South University, Changsha, Hunan Province 410008 China

**Keywords:** Dietary selenium, Diabetes, Cross-sectional study

## Abstract

**Background:**

Selenium is an important trace element for human health. Although numerous epidemiological and interventional studies have examined the association between selenium and diabetes, their findings have been inconclusive. Moreover, no research has specifically focused on the association between dietary selenium and diabetes in the Asian population. The objective of this study was to evaluate the relationship between dietary selenium and diabetes in middle-aged and elderly Chinese adults.

**Methods:**

A cross-sectional study including 5,423 subjects was carried out. The basic characteristics, biochemical test results, and dietary intake were collected from each subject for analysis. The adjusted odds ratio (OR) and the corresponding 95% confidence interval (CI) were used to determine the relationship between dietary selenium intake and diabetes through logistic regression.

**Results:**

The prevalence of diabetes in the study population was 9.7%, and the average level of dietary selenium intake was 43.51 μg/day. The multivariate adjusted OR was 1.52 (95% CI: 1.01 to 2.28, P = 0.04) for the highest quartile of dietary selenium intake in comparison with the lowest quartile. There was a significant positive association between dietary selenium intake and diabetes (P for trend = 0.03).

**Conclusion:**

There was a significant positive correlation between dietary selenium intake and the prevalence of diabetes.

## Introduction

Selenium, which plays a crucial role in human health, is a basic component of selenoprotein, an important enzyme in the body. As suggested by several previous studies, selenoproteins play a functional role in redox homeostasis, thyroid hormone metabolism, and protection from oxidative stress and inflammation [1]. Most selenium-enriched foods are natural foods such as organ meat, seafood, cereals, and crops. The level of selenium intake varies greatly among individuals and populations [2]. For example, it is commonly low in Europe but high in Venezuela, Canada, the United States, and Japan [1,3]. According to a previous study [4], in China the level of selenium intake exhibited huge variance among different population groups.

In view of its important function in protection against oxidative stress, selenium was suggested to play a protective role against type 2 diabetes [5]. However, the association between selenium and diabetes is in fact very complicated. Although numerous epidemiological studies have explored this association, their findings have been inconclusive. Some researchers concluded that a high level of selenium could reduce the prevalence of diabetes [6-8], whereas others suggested that a high level of serum selenium could be related to the increased prevalence of diabetes [9,10]. Meanwhile, the results of the Selenium and Vitamin E Cancer Trial (SELECT) rejected any significant relationship between supplementary selenium and the risk of type 2 diabetes [11]. However, the outcomes of a post hoc analysis of the Nutritional Prevention of Cancer (NPC) trial revealed a significantly increased risk for type 2 diabetes in participants taking supplementary selenium [12].

Previous studies have suggested that the specific association between selenium and diabetes remains unclear. Researchers often focused on the association between diabetes and the selenium concentration in serum, plasma, or nail. Of the limited number of studies thus far to assess the association between dietary selenium and diabetes, one concluded that an increase in dietary selenium intake was associated with an increased risk of type 2 diabetes [13]. It is noteworthy that there was an absence of data focusing on the association between dietary selenium and diabetes in the Asian population. Moreover, the level of dietary selenium intake varied greatly among different population groups because of differences in geographical location and dietary habits.

In view of the current research status, the objective of this study was to assess the average level of dietary selenium intake in the central south area of China, and to examine the association between dietary selenium and diabetes in a large sample from the Chinese population by means of a cross-sectional study.

## Method

### Study population

With the support and promotion of the Chinese government, routine health checkups have become very common in China. In this study, registered nurses interviewed all participants during a medical examination using a standard questionnaire, with the purpose of collecting information on demographic characteristics and health-related habits. The protocol of this study had been approved by the Ethics Committees on Research of the Xiangya Hospital, Central South University (No. 201312459). Subjects were screened according to the following inclusion criteria: (1) aged 40 years or older; (2) undergoing a blood glucose test; (3) availability of all basic characteristics, including age, gender, waist circumference, and body mass index (BMI); and (4) completion of the Semi-Quantitative Food Frequency Questionnaire (SFFQ) about food and drink consumption over the past year and the structured questionnaire containing demographic information and lifestyle habits (smoking, alcohol consumption, and regular exercise). A total of 10,370 subjects aged 40 years or older underwent a blood glucose test during the study period, 10,355 of whom made their basic characteristics available. Ultimately 5,423 subjects completed the SFFQ and the structured questionnaire. The response rate of the SFFQ was 52.4%.

### Dietary assessment

Dietary intake was evaluated using a version of the SFFQ specially designed for the Chinese population. This SFFQ contains 63 food items that are popularly and commonly consumed in Hunan province. The participants were requested to answer how frequently (never, once a month, 2–3 times a month, 1–3 times a week, 4–5 times a week, once a day, twice a day, or more than twice a day) they consumed each food item, and the average amount in grams of consumption (<100, 100–200, 201–300, 301–400, 401–500, and >500) each time they ate during the past year. Color pictures of food samples with labeled weight were provided to help the participants make choices more easily and accurately. The validity of the SFFQ was estimated by comparing the results with the 24-h dietary recall method for the same population. The SFFQ was validated in a subsample of 55 subjects randomly chosen from the study population. The correlation between the SFFQ and the 24-h recalls on the measurement of selenium intake was 0.30. The Chinese Food Composition Table [14] was used to calculate the individual composition of macronutrients and micronutrients of the included food items.

### Blood glucose assessment

All blood samples were drawn after a 12-h overnight fast and were kept at 4°C until analysis. Blood fasting glucose was measured using the glucose oxidase enzyme method. Diabetes was defined as a fasting blood glucose concentration ≥7.0 mmol/L or currently undergoing drug treatment for control of blood glucose.

### Statistical analysis

Quantitative data were expressed as mean ± standard deviation, and qualitative data as a percentage. The selenium intake was classified into four categories based on the quartile distribution in the study population: ≤29.56, 29.57–40.14, 40.15–52.20, and ≥52.21 μg/day. Differences in the continuous data were evaluated by one-way analysis of variance (normally distributed data) or the Kruskal–Wallis H test (non-normally distributed data). Differences in the qualitative data were assessed by the χ^2^ test. The odds ratio (OR) with 95% confidence interval (CI) for the association between selenium intake and diabetes were calculated for each quartile of selenium intake, and the lowest quartile was regarded as the reference category. To calculate the adjusted OR for each quartile of selenium intake, a multivariables model was adopted to perform multivariate logistic analyses. The multivariables model included age, sex, BMI (calculated as weight in kg divided by height in m^2^), activity level, waist circumference, hypertension, alcohol consumption, smoking status, energy intake, fiber intake, and nutritional supplementation status. Tests for linear trends were conducted using logistic regression with a median variable of selenium level in each category. Subgroup analyses were conducted to investigate the potential interaction between selenium intake and sex or BMI. Multivariable adjusted ORs and related 95% CIs were calculated in the sex subgroup (male and female) and the BMI subgroup (BMI ≥25 or BMI <25). All data analyses were performed using SPSS 17.0. P < 0.05 was considered to be statistically significant.

## Results

The basic characteristics according to the quartiles of dietary selenium intake of the study population are shown in Table 1. A total of 5,423 subjects aged 40 years or older (2,882 male and 2,541 female) were included in this study. The average level of dietary selenium intake was 43.51 μg/day, which is close to the recommended nutrient intake (RNI) level of 50 μg/day for the Chinese population [15]. Significant differences were observed across all quartiles of selenium intake in terms of age, sex, education level, employment, BMI, activity level, waist circumference, alcohol consumption, smoking status, nutritional supplementation, energy intake, fiber intake, and fasting glucose. The overall prevalence of diabetes of the target population was 9.7%.

**Table 1:** **Characteristics of the study population according to dietary selenium intake**

n	Se intake				P ^ # ^
	Q1 (lowest)	Q2	Q3	Q4 (highest)	
	1357	1356	1356	1354	-
Median Se intake (μg/day)	22.95	35.07	45.48	62.68	-
Age	53.54 ± 7.76	53.40 ± 7.47	52.89 ± 7.57	52.38 ± 7.27	0.00
Sex (% female)	60.6	52.3	41.9	32.6	0.00
Education level (% with or above high school background)	35.9	44.2	50.8	56.2	0.00
Employment (% manual worker)	29.0	20.3	16.0	15.7	0.00
BMI (kg/m^2^)	24.03 ± 3.28	24.33 ± 3.15	24.50 ± 3.16	24.99 ± 3.06	0.00
Activity level (h/week)	2.25 ± 3.82	2.19 ± 3.42	2.21 ± 3.35	2.42 ± 3.53	0.00
Waist circumference (cm)	81.51 ± 9.10	82.58 ± 8.80	83.10 ± 8.83	84.63 ± 8.68	0.00
Hypertension (%)	32.6	32.7	32.6	33.7	0.92
Drinking (%)	26.2	33.0	36.2	48.3	0.00
Smoking (%)	16.9	21.8	24.9	28.2	0.00
Nutritional supplementation (%)	29.1	35.9	36.4	39.4	0.00
Energy intake (Kcal/day)	1036.84 ± 9.63	1392.87 ± 321.78	1703.74 ± 388.07	2419.71 ± 1010.46	0.00
Fiber intake (g/day)	9.63 ± 6.86	15.00 ± 8.78	19.40 ± 10.68	29.00 ± 18.73	0.00
FG (mmol/L)	5.66 ± 1.52	5.62 ± 1.45	5.75 ± 1.70	5.84 ± 1.83	0.01

Se: selenium, BMI: body mass index, FG: fasting plasma glucose.

Data are mean ± standard deviation, unless otherwise indicated.

^#^P values are for test of difference across all quartiles of selenium intake.

Characteristics of the subjects according to their diabetic status are shown in Table 2. Significant differences were observed between the diabetic and non-diabetic population in terms of age, sex, BMI, waist circumference, hypertension, smoking status, energy intake, fiber intake, and selenium intake.

**Table 2:** **Characteristics of the study population according to diabetic status**

	Diabetic status		P ^ # ^
	Diabetes population	Non-diabetes population	
n	525	4898	-
Age	54.70 ± 7.33	52.87 ± 7.53	0.00
Sex (% female)	35.0	48.1	0.00
Education level (% with or above high school background)	44.8	47.0	0.33
Employment (% manual worker)	18.1	20.5	0.20
BMI (kg/m^2^)	25.68 ± 3.06	24.33 ± 3.17	0.00
Activity level (h/week)	2.39 ± 3.65	2.25 ± 3.52	0.66
Waist circumference (cm)	86.85 ± 8.15	82.54 ± 8.91	0.00
Hypertension (%)	52.6	30.8	0.00
Drinking (%)	39.4	35.5	0.08
Smoking (%)	30.3	22.2	0.00
Nutritional supplementation (%)	31.6	35.6	0.07
Energy intake (Kcal/day)	1752.53 ± 915.48	1625.60 ± 764.22	0.00
Fiber intake (g/day)	19.79 ± 17.77	18.08 ± 13.59	0.02
Se intake (μg/day)	46.76 ± 25.29	43.16 ± 22.35	0.00

Se: selenium, BMI: body mass index.

Data are mean ± standard deviation, unless otherwise indicated.

^#^P values are for test of difference between different diabetic statuses.

The results of the multivariable adjusted association between selenium intake and diabetes are shown in Table 3. With adjustment for age, gender, education level (beyond high school or not), employment (manual worker or non-manual worker), BMI (≥25 or <25), activity level, waist circumference, hypertension, alcohol consumption, smoking status, energy intake, fiber intake, and nutritional supplementation status, the multivariable adjusted OR was 1.52 (95% CI 1.01–2.28; P = 0.04) for the highest quartile of dietary selenium intake in comparison with the lowest quartile (P for trend = 0.03). The results suggested a significant positive association between selenium intake and diabetes. The outcomes of subgroup analysis are listed in Table 3. It can be observed that the significant positive association between selenium intake and diabetes also existed in the subgroup with BMI <25 (P for trend = 0.02). Outcomes from the male subgroup suggested a small positive association between selenium intake and diabetes, but this did not reach statistical significance (P for trend = 0.09). The female subgroup and the subgroup with BMI ≥25 did not show a significant positive association between selenium intake and diabetes (P for trend = 0.35 and 0.47, respectively).

**Table 3:** **Multivariable-adjusted relationship between dietary selenium intake and diabetes in total population, sex subgroup and BMI subgroup**

	Quartiles of Se intake				P for trend
	Q1 (lowest)	Q2	Q3	Q4 (highest)	
Median Se intake (μg/day)	22.95	35.08	45.48	62.69	-
Total population					
*Multivariable adjusted OR	1.00 (reference)	1.10 (0.82, 1.48)	1.32 (0.94, 1.86)	1.52 (1.01, 2.28)	0.03
Sex subgroup					
Male	1.00 (reference)	1.12 (0.74, 1.69)	1.31 (0.84, 2.05)	1.51 (0.90, 2.52)	0.09
Female	1.00 (reference)	1.13 (0.73, 1.75)	1.38 (0.79, 2.41)	1.39 (0.68, 2.86)	0.35
BMI subgroup					
BMI ≥ 25	1.00 (reference)	1.21 (0.77, 1.89)	1.52 (0.91, 2.54)	1.99 (1.09, 3.63)	0.02
BMI < 25	1.00 (reference)	1.00 (0.67, 1.49)	1.16 (0.73, 1.84)	1.20 (0.69, 2.10)	0.47

Se: selenium, OR: odds ratio.

*Multivariable included age, sex, education level (with or above high school background or not), employment (manual worker or non-manual worker), BMI (≥25, <25), activity level, waist circumference, hypertension, drinking, smoking condition, energy intake, fiber intake and nutritional supplementation status.

## Discussion

We conducted a cross-sectional study on a large sample comprising 5,423 middle-aged and older adults in Hunan province, China, the primary objective being to investigate the association between dietary selenium and diabetes. The prevalence of diabetes in the study population was 9.7%, which was very close to the estimated level based on a representative sample of Chinese adults in 2010 (11.6%) [16]. The results indicated that there was a significant positive association between dietary selenium intake and the prevalence of diabetes.

Although several studies have reported an association between selenium intake (dietary or supplementary) and diabetes, their conclusions are inconsistent. Only one study explored the relationship between dietary selenium intake and risk of type 2 diabetes in northern Italy [13], and concluded that increased dietary selenium intake was associated with an increased risk of type 2 diabetes. Meanwhile, some studies focused on the effect of supplementary selenium on diabetes. For example, a randomized, placebo-controlled trial suggested that selenium supplementation may be associated with adverse effects on blood glucose homeostasis in patients with type 2 diabetes [17], while another also observed a significantly increased risk for type 2 diabetes in a population taking a selenium supplement [12]. Nevertheless, the results of the Selenium and Vitamin E Cancer Trial (SELECT) rejected any significant relationship between supplementary selenium and the risk of type 2 diabetes [11]. In addition, a number of epidemiological and interventional studies also reached contradictory conclusions on the relationships between diabetes and serum selenium, plasma selenium, and nail selenium [6-10,12,18-23]. Findings from the Third National Health and Nutrition Examination Survey (NHANES III) [9] and NHANES 2003–2004 [10] both demonstrated a positive association between serum selenium and the prevalence of diabetes. This was confirmed by a previous meta-analysis [24] suggesting a small increased risk of type 2 diabetes with selenium supplementation, although this did not reach statistical significance (pooled relative risk 1.06, 95% CI 0.97–1.15).

The findings of the present study suggested a significant positive association between dietary selenium intake and diabetes, consistent with the conclusions of studies on associations between dietary selenium and diabetes [13], supplementary selenium and diabetes [12], and serum selenium and diabetes [9,10]. Selenium has multiple and complex effects on the human body. In view of its antioxidant capacity, selenium is expected to exhibit an antidiabetic effect. Therefore, some researchers have suggested a protective role for selenium intake, as it could resist oxidative stress-related chronic complications in the progression of diabetes [25-27]. However, the therapeutic range of selenium is relatively narrow, and some selenium compounds could generate toxic reactive oxygen species [8]. Overaccumulation of reactive oxygen species may increase insulin resistance and impair pancreatic β-cell function [28-31]. An increased level of dietary selenium intake may increase the release of glucagon, which can lead to hyperglycemia [32]. In addition, a high level of dietary selenium intake may result in excess expression of glutathione peroxidase 1 (GPx1), which is a type of antioxidant selenoprotein. The high activity of GPx1 can interfere with insulin signaling, which is critical to the regulation of glucose levels and the prevention of diabetes [33]. A review article [3] that comprehensively investigated the association between selenium and type 2 diabetes concluded that this complicated relationship may be explained by the possible harm that occurs both below and above the physiological range for optimal activity of some or all selenoproteins. The present study and a previous one [13] were both conducted on a population with mean dietary selenium intake close to the RNI, but the association in a population with very low dietary selenium intake may be different. Previous review articles suggested that the association between selenium and diabetes might be U-shaped: selenoproteins both below and above the physiological range might become a risk factor for diabetes [1,3,34].

The major strength of this report lies in it being the first to evaluate the association between dietary selenium intake and diabetes in an Asian population. Most previous studies of this relationship have been conducted in the United States or Europe. It is worthwhile demonstrating this association particularly for the Asian population because differences in ethnicity, geographical locations, and dietary habits can potentially affect the results. More importantly, this study is the second to explore the relationship between dietary selenium intake and diabetes using a large-sample, cross-sectional design, and its findings are consistent with those of the former [13]. However, there are also several limitations to this research. First, this cross-sectional study is unable to explain the causal relationship, so further prospective studies are needed to confirm our conclusion. Second, owing to constraint of resources, serum or plasma selenium was not measured for the target population in this study. Study of the relationships between dietary selenium, serum selenium, and diabetes may provide a more comprehensive understanding of this topic. Another potential limitation may lie in the selection bias of the study population. The participants who were undergoing a health examination in the hospital may not represent the general population. However, the large sample size of this study enabled us to examine the association between dietary selenium intake and diabetes using multivariable logistic regression modeling with comprehensive adjustments of confounders. The prevalence of diabetes in the target population was 9.7%, which was very close to the level estimated on the basis of a representative sample of Chinese adults in 2010 [16], therefore suggesting reasonable representativeness of the study population.

## Conclusion

The average level of dietary selenium intake is 43.51 μg/day among middle-aged and older Chinese adults in Hunan province, China. There is a significant positive correlation between dietary selenium intake and the prevalence of diabetes in the target population.

### Consent

Written informed consent was obtained from the patient for the publication of this report and any accompanying images.

## References

[1] Rayman MP (2012). Selenium and human health. Lancet.

[2] Rayman MP (2008). Food-chain selenium and human health: emphasis on intake. Br J Nutr.

[3] Rayman MP, Stranges S (2013). Epidemiology of selenium and type 2 diabetes: can we make sense of it?. Free Radic Biol Med.

[4] Yu SY, Mao BL, Xiao P, Yu WP, Wang YL, Huang CZ (1990). Intervention trial with selenium for the prevention of lung cancer among tin miners in Yunnan, China. A pilot study. Biol Trace Elem Res.

[5] Steinbrenner H, Sies H (2009). Protection against reactive oxygen species by selenoproteins. Biochim Biophys Acta.

[6] Navarro-Alarcon M, Lopez-G DLSH, Perez-Valero V, Lopez-Martinez C (1999). Serum and urine selenium concentrations as indicators of body status in patients with diabetes mellitus. Sci Total Environ.

[7] Kljai K, Runje R (2001). Selenium and glycogen levels in diabetic patients. Biol Trace Elem Res.

[8] Rajpathak S, Rimm E, Morris JS, Hu F (2005). Toenail selenium and cardiovascular disease in men with diabetes. J Am Coll Nutr.

[9] Bleys J, Navas-Acien A, Guallar E (2007). Serum selenium and diabetes in U.S. adults. Diabetes Care.

[10] Laclaustra M, Navas-Acien A, Stranges S, Ordovas JM, Guallar E (2009). Serum selenium concentrations and diabetes in U.S. adults: National Health and Nutrition Examination Survey (NHANES) 2003–2004. Environ Health Perspect.

[11] Lippman SM, Klein EA, Goodman PJ, Lucia MS, Thompson IM, Ford LG (2009). Effect of selenium and vitamin E on risk of prostate cancer and other cancers: the Selenium and Vitamin E Cancer Prevention Trial (SELECT). JAMA.

[12] Stranges S, Marshall JR, Natarajan R, Donahue RP, Trevisan M, Combs GF (2007). Effects of long-term selenium supplementation on the incidence of type 2 diabetes: a randomized trial. Ann Intern Med.

[13] Stranges S, Sieri S, Vinceti M, Grioni S, Guallar E, Laclaustra M (2010). A prospective study of dietary selenium intake and risk of type 2 diabetes. BMC Public Health.

[14] Yang Y. Chinese Food Composition Table*.* Beijing, China:Peking University Medical Press; 2009.

[15] Sun C. Nutrition and Food Hygiene. Beijing, China: People's Medical Publishing House; 2013.

[16] Xu Y, Wang L, He J, Bi Y, Li M, Wang T (2013). Prevalence and control of diabetes in Chinese adults. JAMA.

[17] Faghihi T, Radfar M, Barmal M, Amini P, Qorbani M, Abdollahi M, Larijani B. A Randomized, Placebo-Controlled Trial of Selenium Supplementation in Patients With Type 2 Diabetes: Effects on Glucose Homeostasis, Oxidative Stress, and Lipid Profile. Am J Ther. 2014;21:491-5. 10.1097/MJT.0b013e318269175f 23633679

[18] Czernichow S, Couthouis A, Bertrais S, Vergnaud AC, Dauchet L, Galan P (2006). Antioxidant supplementation does not affect fasting plasma glucose in the Supplementation with Antioxidant Vitamins and Minerals (SU.VI.MAX) study in France: association with dietary intake and plasma concentrations. Am J Clin Nutr.

[19] Gao S, Jin Y, Hall KS, Liang C, Unverzagt FW, Ji R (2007). Selenium level and cognitive function in rural elderly Chinese. Am J Epidemiol.

[20] Coudray C, Roussel AM, Mainard F, Arnaud J, Favier A (1997). Lipid peroxidation level and antioxidant micronutrient status in a pre-aging population; correlation with chronic disease prevalence in a French epidemiological study (Nantes, France). J Am Coll Nutr.

[21] Hughes K, Aw TC, Kuperan P, Choo M (1997). Central obesity, insulin resistance, syndrome X, lipoprotein(a), and cardiovascular risk in Indians, Malays, and Chinese in Singapore. J Epidemiol Community Health.

[22] Akbaraly TN, Arnaud J, Rayman MP, Hininger-Favier I, Roussel AM, Berr C (2010). Plasma selenium and risk of dysglycemia in an elderly French population: results from the prospective Epidemiology of Vascular Ageing Study. Nutr Metab (Lond).

[23] Gao H, Hagg S, Sjogren P, Lambert PC, Ingelsson E, van Dam RM (2014). Serum selenium in relation to measures of glucose metabolism and incidence of Type 2 diabetes in an older Swedish population. Diabet Med.

[24] Rees K, Hartley L, Day C, Flowers N, Clarke A, Stranges S (2013). Selenium supplementation for the primary prevention of cardiovascular disease. Cochrane Database Syst Rev.

[25] Mueller AS, Mueller K, Wolf NM, Pallauf J (2009). Selenium and diabetes: an enigma?. Free Radic Res.

[26] Steinbrenner H, Speckmann B, Pinto A, Sies H (2011). High selenium intake and increased diabetes risk: experimental evidence for interplay between selenium and carbohydrate metabolism. J Clin Biochem Nutr.

[27] Rosen P, Nawroth PP, King G, Moller W, Tritschler HJ, Packer L (2001). The role of oxidative stress in the onset and progression of diabetes and its complications: a summary of a Congress Series sponsored by UNESCO-MCBN, the American Diabetes Association and the German Diabetes Society. Diabetes Metab Res Rev.

[28] Evans JL, Maddux BA, Goldfine ID (2005). The molecular basis for oxidative stress-induced insulin resistance. Antioxid Redox Signal.

[29] Fridlyand LE, Philipson LH (2005). Oxidative reactive species in cell injury: Mechanisms in diabetes mellitus and therapeutic approaches. Ann N Y Acad Sci.

[30] Houstis N, Rosen ED, Lander ES (2006). Reactive oxygen species have a causal role in multiple forms of insulin resistance. Nature.

[31] Oberley LW (1988). Free radicals and diabetes. Free Radic Biol Med.

[32] Satyanarayana S, Sekhar JR, Kumar KE, Shannika LB, Rajanna B, Rajanna S (2006). Influence of selenium (antioxidant) on gliclazide induced hypoglycaemia/anti hyperglycaemia in normal/alloxan-induced diabetic rats. Mol Cell Biochem.

[33] Goldstein BJ, Mahadev K, Wu X (2005). Redox paradox: insulin action is facilitated by insulin-stimulated reactive oxygen species with multiple potential signaling targets. Diabetes.

[34] Rocourt CR, Cheng WH (2013). Selenium supranutrition: are the potential benefits of chemoprevention outweighed by the promotion of diabetes and insulin resistance?. Nutrients.

